# Reviewing the Implication of Aldehyde Dehydrogenases in Male Reproduction: Prospects for New Therapeutic Approaches

**DOI:** 10.3390/ph19040617

**Published:** 2026-04-14

**Authors:** Foteini Gkaitatzi, Ilias Tsochantaridis, Olga Pagonopoulou, Georgia-Persephoni Voulgaridou

**Affiliations:** 1 PEDY—National Primary Health Care Network, 68132 Alexandroupolis, Greece; gkaitatzifoteini@hotmail.com; 2 Department of Molecular Biology and Genetics, School of Health Sciences, Democritus University of Thrace, 68100 Alexandroupolis, Greece; 3 Laboratory of Neurophysiology, Department of Medicine, School of Health Sciences, Democritus University of Thrace, 68100 Alexandroupolis, Greece; opagonop@med.duth.gr; 4 Laboratory of Physiology, Department of Medicine, School of Health Sciences, Democritus University of Thrace, 68100 Alexandroupolis, Greece

**Keywords:** RA metabolism, ALDH activity, spermatogenesis, sperm quality, male infertility

## Abstract

The World Health Organization (WHO) defines infertility as the inability of a couple to conceive after at least 12 months of regular, unprotected sexual intercourse. The male factor appears to be contributing, solely or in combination with other causes, to approximately 50% of all infertility cases. Several etiological factors of male infertility have been identified; however, the exact molecular mechanisms underlying sperm dysfunction are not yet fully understood. Aldehyde dehydrogenases (ALDHs) are multifaceted metabolic enzymes that catalyze the detoxification of several aldehydes, thus acting as antioxidants, while they regulate additional homeostatic functions by contributing to retinoic acid (RA) synthesis. Consequently, they have been identified as crucial factors in various pathogenetic mechanisms. ALDHs hold physiological roles in the testis through supporting the Sertoli cell function, the steroidogenesis in Leydig cells, and the maintenance of sperm integrity. Current evidence supports that dysregulation of specific ALDHs isoforms could be associated with disrupted testicular cell function, including oxidative imbalance and altered RA synthesis. These irregularities could interfere with germ cell development and, subsequently, contribute to decline in reproductive function. In this paper, we are reviewing the role of ALDHs in male reproduction and how their dysregulation could be implicated in male infertility. Unraveling the mechanisms underlying the association of ALDHs with male reproductive function could hold clinical interest regarding the development of novel approaches for enhancing male fertility.

## 1. Introduction

Infertility is defined, according to the World Health Organization (WHO), as the inability of a couple to achieve pregnancy after, at least, 12 months of unprotected, frequent sexual intercourse [1]. It is estimated that infertility has a 17.5% lifetime prevalence and 12.6% period prevalence, with around 1 in 6 individuals experiencing infertility at least once in their lifetime [2]. In approximately 20% of affected couples, infertility is exclusively attributed to the male factor, while male infertility is involved as a cofactor in an additional 30–40% of cases. Consequently, 50% of all infertility cases are associated, to an extent, with reduced male reproductive potential [3,4,5].

Several studies indicate a decline in male reproductive parameters over the last few decades [6,7,8,9]. A meta-analysis by Levine et al. [8] on the global trends in specific sperm variables in studies published during 1973–2018 revealed declines of 51.6% and 62.3% for sperm concentration and total sperm count respectively among men from all continents. Additionally, the authors reported that the decline slope of the sperm concentration and total sperm count is getting steeper in the 21st century [8]. However, while the reduction in the semen parameters is generally accepted, it is not well determined whether this is translated into true reproductive malfunction [10].

Even though the deterioration in male fertility comes with a heavy financial and psychological burden, the etiological factors underlying male subfertility have not yet been fully clarified. This does not come as a surprise considering the multiple genetical, anatomical, endocrinal, metabolic, environmental and lifestyle-related factors associated with reproductive function [11]. Furthermore, in a relatively high proportion of cases (10–20%), male infertility is idiopathic; thus, semen analysis appears with normal parameters, and no specific cause can be identified [5].

Consequently, the management of male infertility is a multifaceted and challenging task, especially in cases where the pathophysiological basis is not well-defined or when multiple casual factors co-exist. Clinical approaches, depending on the causality, include drug and/or supplements administration (e.g., follicle-stimulating hormone (FSH), human chorionic gonadotropin (hCG), antioxidants, micronutrient supplements), changes in lifestyle (e.g., weight loss, increased physical activity, smoking cessation, reduced alcohol consumption), invasive microsurgical procedures (e.g., vasovasostomy, epididymovasostomy, sperm retrieval techniques), and genetic counseling [12,13]. Antioxidants have been shown to limit the negative effects of oxidative stress on sperm function and quality associated with male infertility. Supplementation with specific formulations can improve sperm parameters and fertility outcomes, although these effects vary depending on dosage, duration of administration, and individual health status [14]. While such approaches can serve as supportive treatment for male infertility, they should be applied with caution and with appropriate selection of patients who could potentially benefit.

It is apparent that unraveling the complex and diverse pathophysiological conditions leading to male subfertility as well as identifying novel diagnostic biomarkers and therapeutic targets is of crucial importance. This review highlights the roles of aldehyde dehydrogenases (ALDHs), a superfamily of antioxidant enzymes associated with stem cell phenotype and differentiation, in reproductive homeostasis. We summarize the current data on the involvement of ALDHs in testicular function and explore their potential contribution to the dysfunction leading to male infertility. To our knowledge, this is the first review to summarize the effects of ALDHs on male reproduction by elucidating these mechanisms, which may provide valuable clinical insights and support the development of novel therapeutic interventions.

## 2. Overview of the Aldehyde Dehydrogenase Superfamily

**Figure 1:**
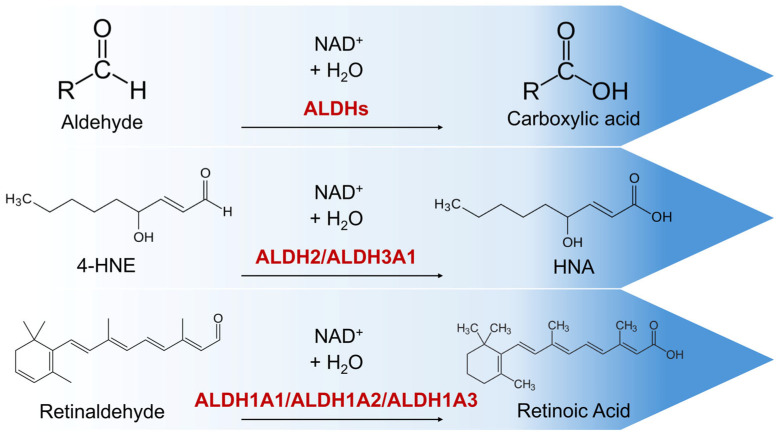
Illustrative examples of ALDHs’ enzymatic activity. ALDHs catalyze the oxidation of aldehydes to their corresponding carboxylic acids. Certain isoforms exhibit strong antioxidant potential by detoxifying toxic aldehydes, by-products of lipid peroxidation, such as 4-HNE, while the ALDH1A subfamily contributes to the synthesis of RA and consequently regulates certain homeostatic mechanisms (e.g., differentiation). ALDHs, aldehyde dehydrogenases; 4-HNE, 4-hydroxynonenal-protein; ALDH1A, aldehyde dehydrogenase 1 family member A; RA, retinoic acid; NAD^+^: Nicotinamide Adenine Dinucleotide.

Members of the ALDH superfamily are encoded by 19 genes and are classified into 11 families and several subfamilies [15,16]. The dual role of ALDHs is that they act as antioxidants by catalyzing the oxidation of a wide variety of reactive and potentially toxic aldehydes to their corresponding carboxylic acids and contribute to the synthesis of important signaling molecules, such as retinoic acid (RA) (via the oxidation of retinaldehyde), which are involved in critical homeostatic processes such as differentiation (Figure 1).

ALDH expression is abundant mainly in tissues with increased metabolic activity and high antioxidant demands including the liver, brain, testis, and ovary. ALDH proteins share a conserved tertiary structure consisting of a catalytic domain with a cysteine residue, a cofactor binding domain for nicotinamide adenine dinucleotide phosphate (NADP^+^), and an oligomerization region required for their quaternary structure formation [17,18]. Individual ALDH isoforms differ in substrate specificity and intracellular localization [19]. The aldehyde dehydrogenase 1 family member A1 (ALDH1A1), aldehyde dehydrogenase 1 family member A2 (ALDH1A2), and aldehyde dehydrogenase 1 family member A3 (ALDH1A3) isoforms, also known as retinal dehydrogenases (RALDHs), catalyze the conversion of retinaldehyde to RA [20,21]. In the testicular tissue, the ALDH1A enzyme family plays a pivotal role in RA biosynthesis within the seminiferous epithelium, a process that is indispensable for proper spermatogenesis [21,22,23]. Aldehyde dehydrogenase 2 family member (ALDH2) has a crucial role in the detoxification of reactive aldehydes such as acetaldehyde and 4-hydroxynonenal (4-HNE) [24]. Enzymes of the aldehyde dehydrogenase 3 family member (ALDH3) family are nicotinamide adenine dinucleotide (NAD+) or NADP+ homodimers and oxidize a wide range of aromatic aldehydes [18]. This family, including aldehyde dehydrogenase 3 family member A1 (ALDH3A1), aldehyde dehydrogenase 3 family member A2 (ALDH3A2), aldehyde dehydrogenase 3 family member B1 (ALDH3B1) and aldehyde dehydrogenase 3 family member B2 (ALDH3B2), is characterized by its unique substrate specificity and shows certain catalytic activity against electrophiles derived from lipid peroxidation, such as 4-HNE, thereby supporting cellular defense against oxidative stress [25,26,27]. ALDH3A1 is predominantly expressed in epithelial tissues [26] and ALDH3A2 participates in the oxidation of long-chain fatty aldehydes, and its mutations lead to Sjögren–Larsson syndrome, an inherited neurocutaneous disorder [27]. Aldehyde dehydrogenase 4 family member A1 (ALDH4A1) is a member of the aldehyde dehydrogenase 4 family (ALDH4), which catalyzes the second step of the proline degradation pathway and is NAD+-dependent in the mitochondrial matrix [28]. Aldehyde dehydrogenase 9 family member A1 (ALDH9A1) in turn catalyzes the NAD+-dependent oxidation of a variety of aldehydes, including the carnitine precursor 4-trimethylaminobutyraldehyde (TMBAL) and the betaine aldehyde [29].

## 3. Retinoic Acid Signaling in Testicular Function

RA is a biological active metabolic product of vitamin A, which, by binding to nuclear receptors such as the retinoic acid receptors (RARs) and the retinoid X receptors (RXRs), regulates cellular differentiation, proliferation and apoptosis through specific signaling pathways [30,31]. Reproductive tissue formation and gametogenesis, including the initiation of meiosis, the regulation of gene expression of steroidogenic hormones, Leydig cell differentiation, and vascular remodeling, appear to depend on the enzymatic synthesis of RA, which is, among others, driven by ALDHs [32]. Genetic and/or functional disorder in the synthesis and signaling of RA can result in reproductive malfunction, including spermatogenesis arrest, testicular atrophy, and infertility [33].

In mammals, spermatogenesis is initiated at puberty [34,35,36]. During embryonic development, bipotential gonadal cells commit to the Sertoli cell lineage in the testis. Primordial germ cells (PGCs) arise in the epiblast and migrate to the developing gonadal ridge [37], where interactions with embryonic Sertoli cells promote seminiferous cord formation [38]. These germ cells subsequently proliferate mitotically to form gonocytes or prospermatogonia, which initially localize to the center of the seminiferous cords and later relocate to the periphery to establish the spermatogonial population in the juvenile testis.

Following puberty, spermatogenesis is a continuous, highly coordinated developmental process that is regulated by RA signaling and cellular interactions within the testicular microenvironment [39], following a species-specific and time-determined program [40]. Complex cellular and molecular interactions, supported by the nutrient environment of the seminiferous tubules, occur to ensure that spermatogenic stem cells (SSCs) enter the differentiation process through spermatogenesis [41]. SSCs represent a specialized population of undifferentiated spermatogonia that, through the property of self-renewal triggered by glial cell-derived neurotrophic factor (GDNF), support the production of germ cells [42]. Upon their commitment to differentiation, the proliferating undifferentiated spermatogonia enter this cyclical program consisting of several distinct stages [40,43,44,45]. During the seminiferous epithelial cycle, RA acts in a pulsatile manner, generating the spermatogenic wave necessary for SSC differentiation, initiation of meiosis, conversion of spermatogonia to spermatocytes and release of structurally mature sperm cells into the lumen of the seminiferous tubules [34,46,47,48]. In the context of asynchronous spermatogenesis, pulsatile RA signaling ensures continuous sperm production through asynchronous germ cell development [49,50]. The coordination of Sertoli and Leydig cells within the testicular microenvironment, along with the basement membrane, is essential for the differentiation process [51]. Sertoli cells, which are regulated by follicle-stimulating hormone (FSH), provide structural and metabolic support, create the blood–testis barrier (BTB) and, through paracrine signaling, regulate germ cell development [52], while Leydig cells support cell function through the production of androgens under the control of luteinizing hormone (LH) [53]. Notably, Sertoli cells synthesize all-trans-retinoic acid (atRA), via ALDH1A1 and/or ALDH1A2 [40], in a pulsatile manner [54]. Dynamic interactions between Sertoli cells and germ cells, accompanied by stage-specific transcriptional changes, are essential for the orderly progression of the seminiferous epithelium cycle [49,55,56]. Additionally, the complementary regulatory involvement of peritubular myeloid cells, resident macrophages and endocrine signaling from the hypothalamic–pituitary–gonadal axis further supports testicular homeostasis [41,57,58,59,60,61].

**Figure 2:**
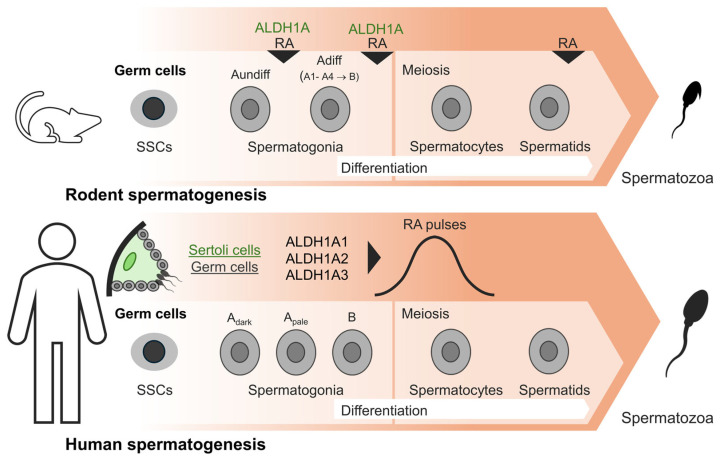
Schematic representation of the involvement of the ALDH1A subfamily in the biosynthesis of RA in the context of rodent and human spermatogenesis. RA regulates key spermatogenesis steps such as the transition of undifferentiated type A spermatogonia into differentiated spermatogonia as well as the entry of spermatocytes in meiosis. RA production is rhythmic within the seminiferous epithelial cycle. SSCs, spermatogenic stem cells; Aundiff, undifferentiated A spermatogonia; Adiff, differentiated A spermatogonia; A_dark_, A-dark spermatogonia; A_pale_, A-pale spermatogonia; B, type B spermatogonia; RA, retinoic acid; A1–A4, type A1, A2, A3, A4 spermatogonia.

In mice, spermatogenesis proceeds in a cyclical and asynchronous way through 12 distinct stages, each defined by characteristic associations of germ cell subtypes [62], whereas in the rat testis, 14 stages (I–XIV) have been described [63]. It should be mentioned that in mice, the process begins around 3–5 days postpartum (DPP) and requires four successive epithelial cycles to generate mature elongated spermatids, with a total duration of approximately 35 days, with the first cycle being shorter, lasting approximately 6 days compared to subsequent cycles lasting 8.6 days [34,55,64,65]. The first wave of spermatogenesis is initiated by Neurogenin-3 (NGN3)-negative prospermatogonia differentiating into A1 spermatogonia, first detected at two days postpartum and progressing through A2, A3, A4, and type B spermatogonia by five days postpartum [34,66]. Undifferentiated spermatogonia, initially referred to as A single (As) spermatogonia, produce paired A (Apr) spermatogonia and then chains of 4 to 32 aligned (Aal) spermatogonia, by mitotic multiplication with Apr and Aal spermatogonia involving undifferentiated cells that retain stem cell properties [47]. RA fluctuations are stage-dependent, with the lowest expression levels observed during stages II–VI and the highest during stages VIII–IX, peaking at stage VIII, leading to the irreversible differentiation of undifferentiated A spermatogonia (Aundiff) to differentiated A1 spermatogonia (A1diff) [67], a key step known as the A to A1 transition [22,49,55,68,69] (Figure 2). The irreversible commitment of Aal spermatogonia to the A1 state initiates the differentiating program that ultimately produces preleptotene spermatocytes [34,70,71,72,73], which subsequently enter meiosis I to initiate the meiotic phase of spermatogenesis.

In primates, undifferentiated spermatogonia consist of A-dark and A-pale populations. In rhesus monkeys, A-pale spermatogonia undergo mitotic division at stage IX of the 12-stage seminiferous epithelium cycle to differentiate into B1 spermatogonia [70]. In the human testis, the seminiferous epithelium is organized into six stages (I–VI), with an estimated cycle duration of approximately 16 days [63] and spermatogonia are divided into dark type A (Ad), pale type A (Ap), and type B subtypes [74] (Figure 2). Concerning human spermatogenesis, it closely resembles that of rodents, as both systems show the progression from spermatogonia to spermatocytes and then to spermatids [56] and several spermatogonia markers are shared between species, with their human homologues potentially correlating with infertility-associated phenotypes [75]. However, despite their morphological similarity to undifferentiated spermatogonia in rodents, the precise identity and function of Ad and Ap cells in human spermatogenesis remain unclear and continue to be debated [76].

## 4. Oxidative Stress in Male Reproduction

**Figure 3:**
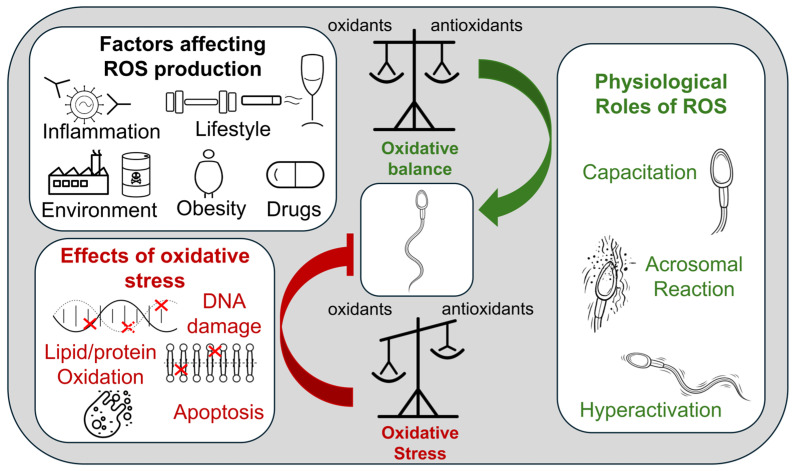
The role of ROS and oxidative stress in male reproduction. Oxidative stress arises from the imbalance between the formation of ROS and the ability of the cellular antioxidant machinery to scavenge them. ROS, in physiological levels, are important for reproductive function; however, in case of imbalance, thus oxidative stress, increased ROS levels result in reproductive malfunction and sperm DNA damage. Increased ROS formation may originate from a variety of endogenous and exogenous stressors. ROS, reactive oxygen species; DNA, deoxyribonucleic acid.

The inability of antioxidant defense mechanisms to balance the production of reactive oxygen species (ROS) results in oxidative stress (Figure 3).

Oxidative stress affects male fertility in approximately 7–10% of the global population and is responsible for 40–50% of idiopathic infertility cases [77]. ROS have a dual role in male reproduction [78]. They are produced normally by spermatozoa through the activity of reduced nicotinamide adenine dinucleotide phosphate (NADPH) oxidase and the reduced nicotinamide adenine dinucleotide (NADH) oxidoreductase system, in the plasma membrane and mitochondria respectively [79], and are required for the key functions of sperm maturation and fertilization. Notably, normal levels of ROS are necessary for a variety of crucial steps for successful fertilization such as sperm hyperactivation, capacitation, and acrosome reaction [78,80]. However, when ROS generation exceeds seminal antioxidant capacity, oxidative stress induces damage and reproductive malfunction. Excess ROS drive lipid peroxidation of the sperm’s polyunsaturated fatty acid (PUFA)-rich membrane and induce protein and deoxyribonucleic acid (DNA) damage, as well as cell apoptosis, impairing sperm motility, viability and fertilizing ability [81]. Spermatozoa are especially vulnerable to such oxidative damage because they have very limited cytoplasmic antioxidants and DNA repair mechanisms, and their membranes are highly enriched in PUFAs [78]. More specifically, elevated ROS levels are associated with mitochondrial dysfunction, sperm membrane lipid peroxidation, sperm DNA fragmentation (SDF) and sperm methylation [14,82]. Regarding lipid peroxidation, cytotoxic aldehydes such as 4-HNE and malondialdehyde (MDA) are produced, which bind to DNA and proteins, causing a decrease in sperm motility [83]. DNA fragmentation resulted from the oxidation of DNA, chromatin compaction, single-strand or double-strand DNA lesions, and activation of endonucleases and caspases [14]. As a result, mitochondria can be affected and therefore produce additional ROS due to dysfunction of their electron transport chain, leading to increased electron leakage. Damaged nuclear DNA can lead to DNA lesions that are transmitted to the embryo during its development [84]. The DNA fragmentation index (DFI) is a useful tool for assessing DNA damage, and a baseline of 30–50% is associated with enhanced infertility and increased pregnancy loss [85,86]. DNA fragmentation has also been linked with adverse outcomes after assisted reproduction by increasing the risk of pregnancy loss [87]. Increased ROS levels can result from either endogenous (e.g., NADPH oxidases, mitochondrial respiration, obesity, inflammation, varicocele) or exogenous (e.g., lifestyle, environmental pollutants, drugs) factors [88]. ROS originating from leukocytes, often due to infection and/or inflammation, can cause DNA fragmentation, protein oxidation, and lipid peroxidation, which, in turn, are linked with impaired sperm quality. Furthermore, mitochondrial dysfunction aggravates oxidative stress, thereby compromising adenosine triphosphate (ATP) production and mitochondrial DNA integrity, leading to energy deficits and reduced sperm motility [88]. Understanding the ROS-related mechanisms linked with infertility is crucial for identifying new approaches as well as improving current therapeutic strategies (e.g., anti-inflammatory and/or antioxidant supplementation) for improving sperm quality and reproductive function [89].

## 5. ALDH and Male Reproduction

### 5.1. ALDHs and RA Signaling

#### 5.1.1. ALDH Enzymes in the Regulation of RA in Male Reproduction

As discussed, RA is vital for spermatogonial differentiation, meiotic initiation, and sperm maturation [40,90,91,92,93]. Proper spatial and temporal regulation of RA levels is critical, as both excessive [94] and insufficient [95,96,97] RA concentrations result in adverse reproductive outcomes. A notable effect of ALDH1A inhibition or vitamin A deficiency is the depletion of testicular RA, resulting in impaired spermatogenesis and male infertility [98,99,100]. Conversely, exogenous RA administration has been shown to downregulate ALDH1 mRNA levels in a dose-dependent manner, indicating the presence of a negative feedback regulatory mechanism [101].

Several studies highlighted the expression of ALDHs in the Sertoli and/or germ line cells, and their consequent contribution to the RA pulses. Regarding their availability, although ALDH1A1 is more abundant in the testis, ALDH1A2 is primarily responsible for RA synthesis during spermatogenesis, while ALDH1A3, though a minor contributor, is expressed in both germ and Sertoli cells [102,103]. Arnold et al. reported that ALDH1A2 activity is mainly localized in spermatogonia, spermatocytes and spermatids, as well as in the peritubular myoid cells (PMCs), with the presence of cellular retinol-binding protein I (CRBP-I) confirming this activity. They also showed that ALDH1A1 is found in Sertoli cells and PMCs, with reduced intratesticular 13-cisRA levels reflecting lower ALDH1A1 expression in men with abnormal semen parameters [102]. It is important to note that apart from its role in testicular physiology, ALDH1A1 also contributes to prostate bud differentiation in the murine urogenital mesenchyme (UGM) and urogenital sinus (UGS) through its involvement in RA synthesis [104,105]. ALDH1A1 is expressed at significantly higher levels in embryonic day 15.5 (E15.5) male UGM compared with female UGM. Inhibition of ALDH1A1 activity with diethylaminobenzaldehyde (DEAB), an ALDH antagonist, markedly reduced the formation of dihydrotestosterone (DHT)-induced homeobox transcription factor Nkx3.1-positive bud in female UGS cultures, whereas RA alone triggered only a limited response, demonstrating that RA can initiate Nkx3.1-positive epithelial specification but does not support full bud development. Furthermore, ALDH1A2 appeared to be involved in the generation of RA pulses in the testis by the Sertoli cells. These pulses activate the expression of gene 8 (Stra8), a key factor required for the initiation of meiosis in spermatogonia [106,107]. ALDH1A3 also supports ALDH1A2 in maintaining RA levels within the testicular microenvironment [108]. In Sertoli cells, ALDH1A1 activity was identified at stages I–VIII and ALDH1A2 activity at stages VII–XII, indicating that during the differentiation process, the initiation of RA production occurs earlier in Sertoli cells than in germ cells [63,108,109,110]. Across different animal species, ALDH1A expression and regulation in the testicular tissue has been shown to vary according to the developmental stage [30,108]. Ιn the canine testis, the expression of ALDH1A1, ALDH1A2 and ALDH1A3 varies from birth to adulthood. During adolescence, where the onset of meiosis and proliferation of germ cells occurs, RA production is mainly dependent on the activity of ALDH1A2, which remains elevated in adulthood, contributing to spermatogenesis [111]. Similarly, an increase in the mRNA and protein levels of ALDH1A2 was observed between the 10th and 20th day postnatally in mouse testis and remained high throughout adult life [112]. Furthermore, in chicken, upregulation of ALDH1A1 has been observed during the differentiation of embryonic stem cells (ESCs) into spermatogenic stem cells (SSCs) [113] and in the teleost fish, Nile tilapia *(Oreochromis niloticus)*, ALDH1A2 expression exhibits an increase prior to the onset of meiosis, indicating its highly conserved role in early spermatogenesis across all vertebrate species [114]. In humans, the first cohort-based study quantifying ALDH1A2 protein levels in testicular tissue from fertile and infertile men was conducted by Amory et al. [115]. They found that ALDH1A2 was significantly reduced in infertile testes and strongly correlated with germ cell populations, particularly more differentiated types such as spermatids, while no association was observed with serum or intratesticular hormone levels. These findings aligned with murine studies identifying ALDH1A2 in germ cells, as shown through immunohistochemical analysis [116]. In contrast, ALDH1A1 and ALDH1A3 levels did not differ between groups and were localized to Leydig and Sertoli cells. The cohort included testicular tissue samples from 43 men, comprising 11 with normal spermatogenesis (primarily with obstructive azoospermia), 20 with Sertoli cell-only syndrome, eight with maturation arrest and four with hypospermatogenesis. Application of a novel mass spectrometry-based peptide assay revealed strong correlations between testicular ALDH1A2 protein levels and germ cell populations. Sertoli cell-only syndrome and hypospermatogenesis were associated with significant reductions in ALDH1A2 levels by 47% and 36% respectively relative to controls, while no significant difference was observed in maturation arrest compared to normal spermatogenesis. Levels were markedly lower in Sertoli cell-only syndrome than in normal tissue, with a moderate decrease in hypospermatogenesis likely reflecting residual germ cells. Elevated serum LH and FSH levels were inversely associated with testicular ALDH1A2, suggesting a compensatory hormonal response. ALDH1A2 expression is regulated by FSH [115], leading to indirect modulation of RA levels and influencing spermatogonial maturation and differentiation [34]. Regarding the function of FSH, in another study in monkey testes, it was found to modify RA signaling, either when administered alone or in combination with LH, significantly increasing the expression of cellular retinoic-binding protein 2 (CRABP2), while the levels of ALDH1A2 and, in the combined treatment, the levels of CRABP1 were reduced [117]. In another study of 19 infertile men with reduced testicular ALDH1A2 levels and low sperm counts, oral administration of isotretinoin, also known as 13-cis-retinoic acid, a retinoid derivative of vitamin A, at 20 mg twice daily for 20 weeks led to an increased sperm concentration and improved morphology [118]. In addition, the human testicular peritubular cells (HTPCs), which form a narrow compartment around the seminiferous tubules and contribute to sperm transport, have recently been characterized by novel features [119]. In situ analysis demonstrated that HTPCs express ALDH1A1 and ALDH1A3 [119]. These cells are capable of producing RA, with RA levels varying in response to fetal calf serum (FCS). While it remains unclear whether HTPC-derived RA directly affects spermatogenesis [55,108], smooth muscle cell (SMC) phenotype [120], or the production of GDNF by Sertoli cells [121], in situ expression of multiple subtypes of RARs supports a potential role in signaling [119].

Referring to the RA pulse, adequate RA levels during the spermatogenic cycle are essential for the proper regulation of spermatogenesis [47]. The production of retinal by retinol dehydrogenase 10 (RDH10) and its subsequent conversion to RA by ALDH1A1, ALDH1A2 and ALDH1A3 [22,23,46,122] results in the pulsatile production of RA along the seminiferous tubules [67]. Kent et al. investigated whether this pattern is expressed in a stage-specific manner [122]. Quantitative analysis showed that ALDH1A1, ALDH1A2, ALDH1A3, and aldehyde dehydrogenase family 8 member A1 (ALDH8A1), an isoform newly identified in the mouse testis, are not stage-specific in adult testes but vary during juvenile development, with ALDH1A2 protein expression exhibiting slight fluctuations before stabilizing in adulthood. Inhibition of ALDH activity resulted in decreased RA levels, increased blood–testis barrier permeability and meiotic deficits. These findings support a broader involvement of ALDH enzymes in spermatogenesis, rather than a role exclusively in regulating RA pulsatility [122,123]. RA signaling is, however, essential for the differentiation of undifferentiated spermatozoa [48,124]. Consistent with this, inhibition of RA biosynthesis with bis(dichloroacetyl)diamine (BDAD), also known as WIN 18,446, an aldehyde dehydrogenase inhibitor [125], caused the arrest of germ cells at the undifferentiated stage in rodent models [126,127]. Quantitative analyses further demonstrated that ALDH1A enzymes are responsible for more than 95% of all-trans RA production in wild-type mouse testes, with ALDH1A2 alone accounting for more than 61% of total atRA synthesis [128]. Surprisingly, trace amounts of atRA persisted in WIN-treated testes even though spermatogenesis had completely ceased [122,129,130], indicating that the A-to-A1 transition likely depends on a threshold level of atRA maintained by fully functional ALDH1A enzymes [129]. In addition, WIN 18,446 combined with exogenous RA supplementation at defined time points synchronized spermatogonial differentiation, enabling the study of their response to RA signals and the isolation of cells at different stages of spermatogenesis, while germ cell separation was simultaneously achieved through fluorescence-activated cell sorting (FACS) [90,124,131]. In cases where an endogenous RA pulse had occurred prior to WIN 18,446 administration, ALDH1A enzyme inhibition did not take effect immediately [129]. In relation to the above, the investigation of the pharmacological inhibition of ALDH1A enzymes is under examination as a potential strategy for reversible male contraception [132]. However, with regard to its clinical application as a reversible [133], non-hormonal male contraceptive [134,135,136,137], the use of WIN 18,446 is limited due to disulfiram-like effects resulting from the inhibition of ethanol-metabolizing enzymes following alcohol consumption [138,139,140,141,142,143]. Ongoing efforts to develop selective inhibitors of testicular RA synthesis that do not interfere with alcohol metabolism [125,133,143,144,145,146,147,148] have recently yielded covalent and non-covalent ALDH1A2 inhibitors, with direct binding studies and X-ray crystallography providing structural insights that guide the rational design of potent and selective candidates for male contraception.

Meiosis is a fundamental biological process that governs both spermatogenesis and oogenesis and ensures the generation of haploid gametes. Abnormalities at various stages of this process can affect reproduction [149] and understanding the regulatory mechanisms underlying this process could be a field of research in the treatment of male infertility [150]. The mechanisms triggering meiotic entry in male germ cells are still not completely understood. Findings from mice with cell-specific deletions of *Aldh1a1*, *Aldh1a2* and *Aldh1a3* have shown that ALDH1A1 activity in Sertoli cells drives the first meiotic wave at puberty, whereas subsequent cycles depend on ALDH1A2 activity within meiotic germ cells [151]. In turn, RA induces pre-meiotic transcriptional changes and *Stra8* expression, which coincides with chromatin remodeling involving histone H3 lysine 27 trimethylation (H3K27me3) at the *Stra8* promoter [152,153].

Recent findings have indicated that RA predominantly initiates the extensive differentiation progress of spermatogonia rather than directly inducing meiotic entry [154,155]. Notably, continuous suppression of RA synthesis following the onset of differentiation using WIN 18,446 did not prevent male germ cells from entering and completing meiosis at the expected time of *Stra8* expression nor did it alter the expression of meiotic genes. In addition, knockout of all three *Aldh1a* genes in Sertoli cells of murine testes disrupted spermatogenesis as A spermatogonia failed to differentiate into A1 spermatogonia [69,123,156], with gross viability maintained [69]. Knockout studies of *Aldh* genes in mice showed that *Aldh1a1*-null mice are viable and fertile [157], *Aldh1a3*-null mice are born alive but die soon after birth from respiratory distress [158] and *Aldh1a2*-null mice die during embryogenesis at day E9.5 to E10.5 [159]. Due to early lethality in *Aldh1a2* and *Aldh1a3,* conditional deletions were used to study ALDH1A function in testes. Either mice with *Aldh1a1*^−/−^ or mice with *Aldh1a2*^−/−^ alone did not exhibit impaired spermatogenesis and a conditional knockout of *Aldh1a2* in germ cells, along with a tamoxifen-inducible knockout model after birth had no major effect on testicular RA levels or fertility, indicating the involvement of enzymes other than ALDH1A [64,129,160]. Spermatogenic differentiation was blocked when all three *Aldh1a* genes were deleted in Sertoli cells, which was restored by a single RA injection [69,156], while the presence of ALDH1A3 alone was not sufficient to maintain spermatogenesis regardless of its compensatory role in different tissues [69]. It has been shown that single deletion of *Aldh1a2* or simultaneous deletion of *Aldh1a1*, *Aldh1a2* and *Aldh1a3* in germ cells has little effect on spermatogenesis and fertility [22,129], while combined deletion in both Sertoli cells and germ cells caused a complete failure that RA injection could not rescue [22]. These findings show that Sertoli cells’ RA pulse is required to initiate spermatogenesis while germ cell RA supports later progression.

#### 5.1.2. Circadian Regulation of RA Biosynthesis by ALDHs in Sertoli Cells and Its Impact on Male Fertility

Sleep disorders negatively affect male reproductive health, with their underlying mechanism being related to the circadian rhythm [161]. Epidemiological evidence suggests that circadian rhythm disruption, a condition frequently observed in shift workers, is associated with male infertility [162,163]. The circadian clock is an endogenous mechanism that governs the 24 h rhythmic variation in the organism’s behavior and physiology, which is regulated by a core set of clock genes that coordinate transcription and translation autoregulatory feedback loops at the cellular level. It consists of a central circadian pacemaker in the hypothalamic suprachiasmatic nucleus (SCN) and peripheral clocks in other brain regions and tissues throughout the body, including the liver, muscle, adipose tissue, and testis, and its genes include circadian locomotor output cycle kaput (*Clock*), aryl hydrocarbon receptor nuclear translocator-like (*Arntl*), period (*Per*)1, *Per2*, *Per3*, cryptochrome (*Cry*)1, and *Cry2*, which are essential for the establishment and maintenance of the circadian rhythm [164,165,166]. When the stimuli from the Zeitgebers are transmitted to the SCN as electrical signals, the circadian rhythm of the clock is orchestrated by genes and proteins that form positive and negative feedback loops [167]. Specifically, CLOCK and BMAL1 form the basic core positive heterodimer complex (CLOCK/BMAL1), which, through its binding to a specific E-box sequence (5′-CACGTG-3′) in the promoters of *Per1*, *Per2* and *Per3*, *Cry1* and *Cry2* and the related clock-controlled genes (*CCGs*), activates their rhythmic transcription [168,169,170,171]. Liu et al. [172] recently found that RA synthesis appears to be under circadian control. Their research showed that the circadian clock within Sertoli cells plays an essential role in synchronizing spermatogenic differentiation and supporting fertilization through the regulation of RA biosynthesis and receptor expression. The expression of genes related to RA signaling peaks in the evening or early night, suggesting a rhythmic pattern of endogenous RA activity in vertebrate testes. Time-series single-cell RNA sequencing (scRNA-seq) further revealed that *aldh1a2* displays rhythmic expression specifically in Sertoli cells, indicating that both core circadian clock genes and their downstream targets may oscillate in a cell type-specific manner. In both zebrafish and mouse testes, a temporal pattern of gene expression related to spermatogenesis and sperm function has been observed. Consistent with this, quantitative real-time reverse transcription PCR (qRT-PCR) analysis in zebrafish testes confirmed that the rhythmic expression of these genes is restricted to Sertoli cells and is abolished in *clock1a* knockout mutants. ScRNA-seq identified the co-expression of *aldh1a2* with circadian clock-regulating genes such as period 1b (*per1b*) and *clock* in Sertoli cells of zebrafish, mice and humans, suggesting the existence of an evolutionarily conserved mechanism for the circadian regulation of RA synthesis. Moreover, co-expression of *Clock* with *aldh1a1* was also detected at the single-cell level, and immunohistochemical (IHC) staining demonstrated the colocalization of brain and muscle ARNT-like protein 1 (BMAL1) and ALDH1A2 proteins in mouse Sertoli cells. Genomic analysis revealed the presence of E-box enhancer elements in the first introns of *aldh1a2* and retinoic acid receptor gamma a (*rarga*), which were shown through luciferase reporter assays to be transcriptionally activated by brain and muscle ARNT-like 1 *bmal1b* and *clock1a* genes, and repressed by the Cryptochrome-1ab (*Cry1ab*) gene. All these findings from the above research study [172] propose a spatially and temporally coordinated model of RA synthesis within the testis mediated by ALDH1A1 and ALDH1A2 activity. RA availability is aligned with germ cell development and highlights the role of ALDH-regulated RA production in maintaining male fertility, particularly under conditions of circadian rhythm disruption [164]. It should be noted that stress, associated with shift work, may also indirectly contribute to circadian rhythm disruption through alterations in cortisol secretion, potentially leading to inflammation and oxidative stress. This represents an interesting potential mechanism of reproductive dysregulation, including possible effects on RA signaling, which warrants further investigation.

### 5.2. ALDHs and Oxidative Status

#### 5.2.1. Metabolic and Redox Functions of ALDH2 and Its Impact on Spermatogenesis

**Figure 4:**
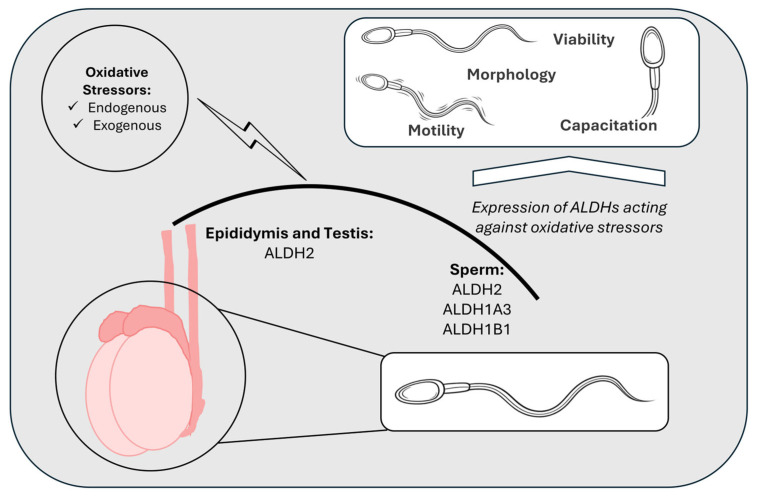
ALDHs protect sperm viability and functionality from a variety of oxidative stressors through their antioxidant and metabolic activities. The expression profile of ALDH isoforms presented here is representative for mammalian species. Among these, ALDH2 is abundantly expressed in the testis and epididymis. ALDH1A3, ALDH1B1 and ALDH2 isoforms are also detected in ejaculated spermatozoa, and ALDH2 likely protects mitochondrial function in OXPHOS-dependent cells while supporting sperm quality. ALDHs, aldehydes dehydrogenases; ALDH1A3, aldehyde dehydrogenase 1 family member A3; ALDH1B1, aldehyde dehydrogenase 1B1; ALDH2, aldehyde dehydrogenase 2; OXPHOS, oxidative phosphorylation system.

ALDHs, apart from their role in RA synthesis, appear to have a crucial role in the protection of spermatozoa functionality (e.g., motility, capacitation, acrosomal reaction) through their detoxifying activities and this becomes even more important under the influence of certain endogenous and exogenous sperm stressors (e.g., inflammation, alcohol consumption, environmental pollutants) (Figure 4).

It is known that ALDH1A1 and mitochondrial ALDH2 metabolize acetaldehyde to acetic acid during alcohol breakdown. The ALDH2*2 allele, which is highly prevalent in East Asian populations, results from a Glu487Lys substitution that inactivates the enzyme, leading to acetaldehyde accumulation after alcohol consumption and causing the characteristic facial flushing response [173]. In addition to ALDH2 deficiency, one of the most common inherited conditions affecting up to 40% of the Asian population and responsible for the Asian Hot Flash Syndrome [174], several findings also support its role in reproduction [175], with differences in sperm quality having been observed between ethnicities, and the involvement of different polymorphisms in these variations remaining under investigation [176,177]. During ethanol (EtOH) metabolism, acetaldehyde is generated not only by cytochrome P450 2E1 (CYP2E1) [178] but also by ALDH within the epididymis [179,180]. Spermatozoa from ALDH2 knockout mice have shown increased sensitivity to the toxicity of ethylene glycol monoethyl ether [181,182]. In the study of Taoto et al. [183], EtOH-treated rats showed histological alterations in the cauda epididymis and seminal vesicle, including epithelial thinning, fibrosis and increased apoptosis. However, in another study, selective knockdown of ALDH2 in germ cells did not affect spermatogenesis nor decrease testicular RA levels in mice [129]. Knockdown of ALDH2 in mice using CRISPR/Cas9 technology led to reduced seminiferous tubule thickness, reduced germ cell number, impaired spermatocyte development and impaired acrosome formation, resulting in oligoasthenoteratozoospermia, although morphological abnormalities in the head and tail remained unchanged [184]. These alterations were accompanied by elevated oxidative stress markers such as 4-HNE, 3-nitrotyrosine (3-ΝΤ) and MDA in both the testis and epididymis. In pigs, a recent quantitative proteomic analysis revealed that Meishan pigs exhibit higher ALDH2 expression than the less fertile Duroc breed [185]. Greenberg et al. [186] examined sperm parameters in relation to alcohol consumption in individuals carrying the ALDH2*2 polymorphism. In a cross-sectional study including 112 East Asian men, 40.2% of whom were ALDH2*2 carriers, alcohol consumption was found to be associated with reduced total and progressive sperm motility among carriers, whereas no adverse effects were observed in those with little or no alcohol consumption, indicating that the effect was dependent on alcohol intake. In another study, Le et al. [187] demonstrated that ethanol exposure has a genotype-specific effect on sperm motility. Sperm from individuals with the heterozygous (GA) or homozygous mutant (AA) ALDH2 genotype exhibited a more pronounced reduction in total motility following ethanol exposure. These findings suggest that ALDH2 deficiency may increase vulnerability due to ethanol exposure causing sperm dysfunction, underscoring its potential role in male fertility and the importance of further investigation, especially given that over 600 million people worldwide carry an ALDH2 mutation.

#### 5.2.2. ALDHs in Sperm Motility and Capacitation Across Fertilization

Data on the role of ALDHs in sperm functionality arise mainly from studies conducted on stallions (adult male horses) and other mammals. Specifically, proteomic profiling of stallion seminal plasma has revealed molecular pathways associated with sperm motility with evidence suggesting that reduced ALDH expression may contribute to the decreased motility observed in infertile stallions [182,188]. The study by Gibb et al. [182] demonstrated the role of ALDH in protecting stallion sperm from oxidative stress induced by 4-HNE, as well as the involvement of glutathione S-transferase (GST) in the detoxification of aldehydes in vitro. In human sperm, adenosine triphosphate (ATP) production is mainly based on glycolysis [189], while in stallion sperm, it is highly dependent on oxidative phosphorylation (OXPHOS) [190,191], resulting in the higher production of reactive oxygen species (ROS) and lipid peroxidation products such as 4-HNE. This allows faster motility than in humans [190], but leads to oxidative damage and rapid loss in vitro [192], with initial activity maintained due to ALDH- and GST-mediated detoxification mechanisms [193,194]. ALDH1A3, ALDH1B1 and ALDH2 were isolated from ejaculated sperm. Among them, ALDH2 identified as a prognostic factor of fertility, possibly by protecting mitochondria, resulting in no loss of motility despite 4-HNE accumulation [190]. This is an evolutionary adaptation to oxidative stress specific to the stallion, which was also reinforced by the observation that upon exogenous administration of 4-HNE, ALDH remained in the midpiece, while 4-HNE adducts amassed in the post-acrosomal region in contrast to human sperm where 4-HNE is predominantly located in the midpiece [195,196]. Further, ALDH inhibition was found to increase 4-HNE levels in viable spermatozoa and significantly reduce both total and progressive motility after 24 h [197,198]. In another study of cryopreserved stallion semen, proteomic profiling revealed that cryopreservation disrupts sperm metabolism and redox balance, causing a significant decrease in antioxidant proteins such as mitochondrial superoxide dismutase 2 (SOD2) and ALDH2 [199], with lipid peroxidation products such as 4-HNE accumulating extensively during this process [200,201]. It has also been demonstrated in the study by Akbarinejad et al. that Activator of mitochondrial aldehyde deydrogonase (Alda-1), an activator of ALDH2, helps preserve mitochondrial function in equine spermatozoa during 72 h cooled storage, leading to enhanced ATP production, motility, and viability [202]. In addition, studies on water buffalo (*Bubalus bubalis*) bulls have shown that ALDH is highly expressed in the spermatozoa of high-fertile (HF) bulls [203], while ALDH2 is highly abundant in extracellular vesicles (EVs) derived from seminal plasma and plays an essential role in sperm motility [204]. During bovine sperm capacitation, several processes occur, such as cytoskeleton and membrane reorganization, vesicle transport, guanosine triphosphate (GTP) binding and redox regulation. This recruits several enzymes, including mitochondrial ALDH2 [205]. Mitochondrial ALDH2, by supporting sperm progressive motility through the elimination of electrophilic aldehydes [182], may further contribute to sperm capacitation and sperm–oocyte interaction via its association with testis-specific angiotensin-converting enzyme (tACE) in the post-acrosomal region and principal piece. tACE, an isozyme of somatic angiotensin-converting enzyme (sACE), regulates capacitation, acrosome reaction, and zona pellucida binding, relocating to the post-acrosomal region after capacitation [205]. In the same study, STRING analysis did not reveal any interaction between ALDH2 and the tACE network in *Bos Taurus*, in contrast to mass spectrometry results, possibly due to limited functional evidence. These findings raise the need for further isoform-specific studies across species, using flow cytometry and the Aldefluor system, especially regarding the effect of oxidative stress on human sperm function [206] and as the beneficial effects of Alda-1 could also affect sperm function in other species, including humans [196].

### 5.3. Environmental Modulations of ALDH Function in Male Reproduction

Environmental toxicants are increasingly recognized as major contributors to male infertility, with compounds such as heavy metals and plasticizers implicated in reproductive system damage. This damage is further influenced by genetic and lifestyle factors. Autophagy-related gene 5 (*Atg5*) contributes to RA biosynthesis in the testis, as Sertoli cell-specific *Atg5* ablation leads to decreased testicular RA and ALDH1A1 levels [207]. In the study by Xiong et al. [207], mice with Sertoli cell-specific knockout of *Atg5* were used to investigate how loss of autophagy influences cadmium (Cd)-induced testicular damage, a widespread dietary and waterborne contaminant that disrupts germ cell development and spermatogenesis. Autophagy, a cellular degradation and recycling process, supports Sertoli cell function and communication with germ cells, and its absence through *Atg5* deficiency intensified the Cd-driven reduction in RA and its key synthesizing enzymes ALDH1A1 and ALDH1A2 in the testes, while overexpression of Wilms tumor 1 (*Wt1*) rescued the Cd-induced decline in ALDH1A1. A comparable reduction in ALDH1A1 expression has been observed in primary Sertoli cells exposed to 3-monochloropropane-1,2-diol (3-MCPD) [208]. In another study by Weng et al., exposure of C57BL/6 male mice to ethyl tertiary butyl ether (ETBE) showed that while only high concentrations impaired sperm motility and increased DNA damage in wild-type animals, even low concentrations caused testicular atrophy, reduced sperm counts, and severe genotoxicity in ALDH2 knockout and heterozygous mice. These results suggest that ALDH2 deficiency markedly increases susceptibility to ETBE-induced reproductive toxicity through the accumulation of acetaldehyde [209]. Additionally, chemoproteomic investigation of rodent testicular toxicity induced by the covalent Bruton’s tyrosine kinase (BTK) inhibitor N-(3-(5-fluoro-2-(4-(2-methoxyethoxy)phenylamino)pyrimidin-4-ylamino)phenyl) acrylamide (CC-292), a compound developed for hematologic cancers and inflammatory diseases, revealed its off-target interaction with ALDH1A1 and ALDH1A2 [210]. Using a biotin-labeled analog (292TC) and click chemistry-based proteomic profiling of testis homogenates, these enzymes were identified as covalent binding targets in both mice and rats. Molecular modeling suggested that CC-292 may bind to the NAD^+^ and retinal sites of human ALDH1A2, posing a potential reproductive risk and supporting its redesign to produce BTK inhibitors that do not interact with ALDH1A1 or ALDH1A2. Also, it has been shown that N-methylthioltetrazole, a toxic component of the β-lactam antibiotic cefamandole, inhibits aldehyde dehydrogenase activity and disrupts spermatogonial proliferation [211]. Furthermore, in the sperm of smokers, altered DNA methylation at specific CpG sites in the *Aldh3b2* gene has been associated with reduced gene expression and negatively correlated with sperm quality parameters [212], suggesting that epigenetic modifications of key genes may contribute to smoke-induced impairments in sperm function [213].

As a key regulator of testicular homeostasis, the testicular immune microenvironment (TIM) becomes vulnerable when challenged by such contaminants, potentially leading to reproductive toxicity [214]. Macrophages (TMΦ) in both the interstitial (iTMΦ) and peritubular (pTMΦ) compartments of the mice testis have been shown to participate in the local regulation of RA signaling, partly through the expression of ALDH1A2 in both populations to facilitate spermatogonial cell proliferation and differentiation [215,216,217]. Their functional relevance is further supported by findings that the transient ablation of testicular macrophages impairs spermatogonial stem cell (SSC) differentiation through the disruption of colony-stimulating factor receptor (CSF1) and RA expression [41,57].

### 5.4. Additional Aspects of Selected ALDH Isoforms in Spermatogenesis and Male Reproductive Function

The study of another ALDH superfamily member, ALDH4A1, by Xiao et al. [218] revealed its essential role in mitochondrial proline metabolism and male fertility. CRISPR-Cas9-mediated knockout of *Aldh4a1* in mice resulted in normal spermatogenesis but defective sperm maturation, characterized by impaired motility, abnormal morphology, elevated spontaneous acrosome reaction [219], and ultrastructural abnormalities in sperm mitochondria and neck. Based on the finding that oxidative stress rises with aging in the testis of mice as a result of decreased levels of antioxidant enzymes, Yen and Curran determined the gene expression of proline dehydrogenase (*Prodh*) and *Aldh4a1*, two proteins associated with proline catabolism in the testes of young and middle-aged mice. They reported relatively similar levels of *Prodh* expression in the two different age groups, while middle-aged mice exhibited lower expression levels of Aldh4a1 in comparison to the younger mice [220]. Furthermore, both *Prodh* and *Aldh4a1* expression were at comparable levels in the liver of the examined groups, suggesting a tissue-specific regulation of these genes in the male reproductive systems. A reduction in ALDH4A1 could potentially lead to pyrroline-5-carboxylic acid (P5C) accumulation, and consequently to sperm dysfunction, as observed in *C. elegans* [221]. Additionally, due to its evolutionary conservation, *Aldh4a1* is a promising candidate for the genetical diagnosis of male infertility.

Huang et al. [222], in their study on Ten-eleven 1 (*Tet-1*) deficiency in translocation and premature reproductive aging, observed that there was a decrease in ALDH3B1 expression accompanied by a decrease in *Tet1* promoter occupancy in *Tet1* knockout (*Tet1*^−/−^) spermatogonia. They used Integrative Genomics Viewer (IGV) to analyze 5-hydroxymethylcytosine (5hmC) enrichment at the *Aldh3b1* promoter in both type A and type B spermatogonia, with stronger signals in the coding exon, indicating a positive regulatory role of 5hmC. Reduced ALDH3B1 expression in aged or *Tet1*^−/−^ spermatogonia likely impairs retinol metabolism and decreases entry into meiosis. In addition, Laqqan et al. [223] reported that subfertility was linked to altered DNA methylation at CpG sites within the *Aldh3b2* and prostaglandin I2 receptor (*PTGIR*) genes, as identified using the Illumina HumanMethylation450 BeadChip (450K) array [224].

In the study by Pini et al. [225], comparative proteomic analyses across vertebrates revealed a conserved molecular framework underlying sperm function, with a core set of proteins involved in key processes such as energy metabolism and fertilization. Among these, ALDH7A1, a metabolic enzyme that affects sperm motility, not previously linked to sperm development [226], was identified as conserved across species.

Proteomic analysis of testicular interstitial fluid (TIF) in men with azoospermia revealed that proteins associated with redox regulation, including 4-trimethylaminobutyraldehyde dehydrogenase (TMABA-DH), also known as ALDH9A1, were upregulated in cases with successful sperm retrieval compared to unsuccessful ones [227]. These findings implicate oxidative stress as a key determinant of retrieval outcomes and suggest that reducing testicular oxidative stress may enhance sperm recovery. Further, ALDH9A1, its human natural killer 1 (HNK1)-modified form, and other HNK1 glycoproteins appear to contribute to sperm negative selection in the utero-tubal junction (UTJ), with acrosome-reacted sperm localizing to protein-rich regions as mating enhances carbohydrate sulfotransferase 10 (CHST10) activity in the oviductal mucosa and drives the HNK1-dependent formation of acidic ALDH9A1 and four and a half LIM domains protein 1 (FHL1) variants in the same areas [228]. In the study by Fábrega-Guerén et al. [228], ALDH9A1 was reported for the first time on the sperm surface and in the acrosome in vivo, although it is first necessary to verify whether it is synthesized by the sperm. Probably, ALDH9A1 in close contact with sperm may regulate spermine levels, as it has been shown to be involved in spermine metabolism and Gamma-aminobutyric acid (GABA) production. Sperm with low spermine content reaching the UTJ could contact ALDH9A1-rich regions, increasing spermine above a critical threshold and triggering spontaneous acrosome reaction (sAR) or capacitation followed by AR due to GABA in the oviductal mucosa.

### 5.5. ALDH Enzymes as Functional Markers and Regulators of Spermatogonial Stem Cell (SSC) Function in Male Fertility Restoration

The generation of gametes from stem cells has emerged as a promising strategy to overcome infertility. Throughout the reproductive life span, continuous sperm production relies on the self-renewal and differentiation capacity of SSCs [229]. The ability to understand, manipulate and culture SSCs in vitro has opened new avenues in reproductive medicine and fertility preservation [230]. Especially, it has been shown that murine SSCs spontaneously transform into pluripotent embryonic stem cells (ESCs) under specific culture conditions [231,232]. Tapia et al. [233] and Chikhovskaya et al. [234] demonstrated that “embryonic-like colonies” derived from SSCs resemble mesenchymal stem cells (MSCs) rather than ESCs, suggesting that these colonies may originate from a subpopulation of mesenchymal progenitors (MPs) present in testicular cultures. SSCs have also been shown to be closely related to very small embryonic stem cells (VSELs). VSELs are characterized by their small size and expression of markers found in progenitor germ cells (PGSs). Although they rarely divide, they are known to survive gonadotoxic insults, such as chemotherapy, and serve as a reserve source for SSCs through asymmetric division [235]. It is important to mention that VSELs spontaneously differentiate into gametes under in vitro conditions, suggesting a potential role in fertility restoration therapies, especially after gonadotoxic treatments [236,237,238,239]. Their ability to regenerate stem cells opens new avenues in reproductive medicine [240,241], while the contribution to defining the factors that regulate SSC maintenance and self-renewal is important for understanding idiopathic infertility and developing therapeutic strategies for restoring fertility in patients receiving chemotherapy, especially during prepubertal development [242]. This evolving perspective in the field of oncofertility challenges the current necessity of germ cell or testicular tissue cryopreservation prior to cancer therapy and in the future, this approach may simplify fertility preservation strategies and improve outcomes for cancer patients [243,244,245]. Various markers contribute to the isolation of SSCs, such as ALDH, Nestin and Musashi-1, which are stemness-related markers and do not involve transcription factors or surface proteins [246]. Kanatsu-Shinohara et al. [247] developed a method for isolating SSCs among the ALDH-negative subpopulation of mouse testicular cells expressing CD9 or CDH1 by detecting the enzymatic activity of ALDH using the Aldefluor reagent. This enables the selective identification and enrichment of SSCs that are located in the basal compartment of the seminiferous epithelium where they adhere to the basement membrane [248]. Also, the isolation and further study of MSCs can be achieved through the detection of the high levels of ALDH observed in them [249]. Regarding fertility in prepubertal cancer patients after chemotherapy, it was found that germ cell depletion in recipient testes disrupted RA homeostasis and impaired spermatogenesis in transplanted donor tissue, possibly and partly due to reduced *Aldh1a2* and sustained *Cyp26b1* expression, while RA treatment restored spermatogenesis, suggesting that combining testis transplantation, RA supplementation, and microinsemination could be an effective strategy for fertility recovery in this patient group [39].

Although ALDH enzymes are commonly used as SSC markers, evidence from a study by Xu et al. [250] indicates that they also actively participate in the regulation of germ cell differentiation, with fatty acid oxidation (FAO) playing a key role during embryonic days E13.5–E15.5, when male germ cells progressively enter mitotic arrest alongside increasing the expression of carnitine palmitoyltransferase 1A (CPT1A). This is a mitochondrial transmembrane enzyme that allows fatty acids to enter the mitochondria, thereby controlling the rate-limiting step for the entire pathway. To investigate the importance of this pathway, pregnant mice were treated with etomoxir, a CPT1A inhibitor, during the same developmental period. This resulted in FAO inhibition leading to reduced acetylation of histone H3 at lysine 27 (H3K27ac) and downregulation of male differentiation-specific genes, resulting in premature PGC exit from mitotic arrest. Quantitative real-time PCR (qPCR) analysis of embryonic testes revealed increased expression of ALDH5 and ALDH9A1, along with enoyl coenzyme A delta isomerase 1 (ECI1), at E15.5 compared with E13.5, indicating their functional involvement in FAO-related metabolic regulation, which is essential for the proper differentiation of male germ cells. Through their study, they hypothesized that the inhibition of FAO during embryonic development may affect the normal energy metabolism of Sertoli cells, leading to increased apoptosis, resulting in a disruption of the microenvironment in the testes after birth and a possible increase in spermatogonia apoptosis.

## 6. Conclusions and Future Perspectives

**Figure 5:**
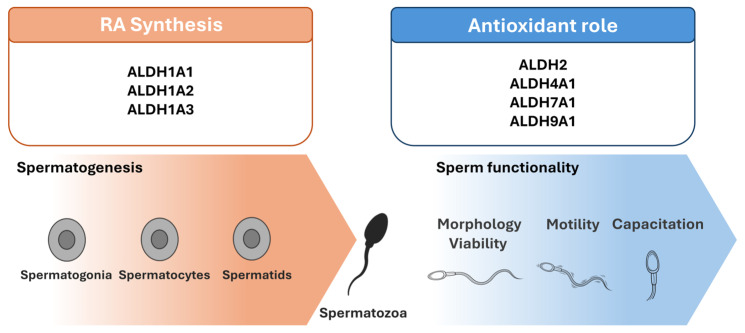
Schematic of the main roles of ALDHs on male reproduction.

ALDH isoforms contribute to male reproductive health through two fundamental and interconnected functions: RA synthesis and cellular antioxidant defense. This dual functionality underscores the importance of ALDHs in the maintenance of reproductive homeostasis and highlights their broad implications for male fertility (Figure 5).

Through their involvement in RA synthesis, ALDHs possess a crucial role in regulating spermatogenesis. They are expressed in both Sertoli and germ cells and, along with other metabolic enzymes, contribute to the creation of the RA pulses that drive spermatogonial differentiation and entry into meiotic division. Emerging evidence further supports that circadian rhythms may orchestrate these RA pulses through the temporal regulation of ALDH1A isoform expression, mainly, in Sertoli cells. Furthermore, ALDHs, with ALDH2 being the most well studied, appear to be significant for maintaining the functionality of spermatozoa by protecting against endogenous and exogenous oxidative stressors. Decreases in ALDH activity, whether due to environmental toxicants, genetic variants or epigenetic dysregulation, have been consistently associated with impaired sperm parameters.

Collectively, a vast amount of evidence supports the significance of ALDHs in reproductive function, through multiple mechanisms, across multiple mammalian species (Table 1).

This suggests that ALDHs may represent potential biomarkers for infertility diagnosis and possible targets for therapeutic interventions. However, more studies are required to elucidate: (i) the precise processes through which ALDHs coordinate RA signaling and redox balance and (ii) the isoform-specific contributions underlying these processes in humans. This is important, considering that current evidence is, to a high proportion, derived from studies in animal models. Additionally, investigation of other isoforms, such as the mitochondrial Aldehyde Dehydrogenase 1 Family Member L2 (ALDH1L2), which regulates NADPH for ROS detoxification and ATP production in sperm, despite evidence suggesting that its loss does not affect male fertility [251] and Betaine aldehyde dehydrogenase (BADH), which supports sperm motility and health through betaine synthesis [252], could provide new evidence and contribute further to the progress in this field.

## Figures and Tables

**Table 1:** Overview of ALDH isoforms associated with male infertility.

ALDH Isoform	Main Mechanism	RA Synthesis	Antioxidant Role	Main Location	Ref.
ALDH1A1	Contributes to retinoic acid (RA) production, which regulates spermatogonial differentiation and the initiation of meiosis during spermatogenesis	Yes (Retinal → RA)	Oxidation of reactive aldehydes such as acetaldehyde and lipid peroxidation-derived aldehydes	Sertoli cells and peritubular myoid cells of the testis. Also detected in Leydig cells and other somatic cells of the seminiferous epithelium	[22,23,40,63,64,69,102,108,109,110,111,113,115,122,123,128,129,151,156,159,207,208,209,210]
ALDH1A2	Primary enzyme for RA synthesis in the testis, generating RA pulses that trigger spermatogonial differentiation and meiotic entry	Yes (primary testicular RA producer, accounts for a large fraction of atRA synthesis)	Minor direct antioxidant role (retinal oxidation to retinoic acid)	Primarily expressed in germ cells and in Sertoli cells, peritubular myoid cells and interstitial and peritubular macrophages	[22,23,40,63,64,69,102,106,107,108,109,110,111,112,114,115,116,122,123,125,128,129,151,156,210,215,216,217]
ALDH1A3	Supports local RA production and maintenance of RA signaling in the seminiferous epithelium, contributing to germ cell differentiation	Yes (contributes to RA synthesis but usually less than ALDH1A2)	Minor detoxification activity toward reactive aldehydes	Expressed in Sertoli cells and germ cells in the seminiferous epithelium	[22,23,40,64,69,102,103,108,111,115,122,123,128,129,156]
ALDH2	Maintains sperm mitochondrial function and motility by detoxifying acetaldehyde and lipid peroxidation products and may further contribute to acrosome formation, sperm capacitation and sperm–oocyte interaction	No	Major mitochondrial detoxification enzyme for acetaldehyde and lipid-peroxidation products (e.g., 4-HNE, malondialdehyde)	Predominantly localized in the mitochondria of spermatozoa, and also present in testicular tissue, epididymis and sperm	[144,174,175,176,177,179,180,181,182,183,184,186,187,190,199,202,204,205,209]
ALDH4A1	Mitochondrial proline metabolism → affects mitochondrial integrity & sperm maturation, motility, morphology and spontaneous acrosome reaction	No	Indirect antioxidant role through mitochondrial proline metabolism	Localized primarily in mitochondria of testicular cells and spermatozoa	[28,218,219,220]
ALDH7A1	Conserved across species. Aldehyde metabolism affecting sperm metabolic homeostasis and sperm motility	No	Detoxifies α-aminoadipic semialdehyde (a-AASA) and other aldehyde intermediates involved in breakdown of lysine	Identified in spermatozoa and testicular tissue	[225,226]
ALDH8A1	Possible role in testicular aldehyde metabolism and metabolic regulation during spermatogenesis	Possible (not confirmed)	Limited evidence for antioxidant activity	Detected in testicular tissue, particularly in mouse testis, according to proteomic studies	[122]
ALDH9A1	FAO-related metabolic regulation for proper differentiation of male germ cells, biogenic aldehyde metabolism and redox balance and triggering spontaneous acrosome reaction (sAR) or capacitation through the increase in spermine above a critical threshold	No	Oxidation of γ-trimethylaminobutyraldehyde and other biogenic aldehydes contributing to carnitine synthesis and may regulate spermine levels, through spermine metabolism and Gamma-aminobutyric acid (GABA) production	Found in testicular interstitial fluid, on the sperm surface and in the acrosome in vivo	[29,227,228,250]

## Data Availability

No new data were created or analyzed in this study. Data sharing is not applicable.

## Abbreviations

The following abbreviations are used in this manuscript:

WHO	World Health Organization
ALDHs	Aldehyde dehydrogenases
RA	Retinoic acid
FSH	Follicle-stimulating hormone
hCG	Human chorionic gonadotropin
4-HNE	4-hydroxynonenal-protein
SSCs	Spermatogenic stem cells
GDNF	Glial cell-derived neurotrophic factor
atRA	All-trans-retinoic acid
NGN3	Neurogenin-3
ROS	Reactive oxygen species
MDA	Malondialdehyde
ATP	Adenosine triphosphate
PMCs	Peritubular myoid cells
UGM	Urogenital mesenchyme
UGS	Urogenital sinus
DHT	Dihydrotestosterone
